# Time-series analysis of geographically specific monthly number of newly registered cases of active tuberculosis in Japan

**DOI:** 10.1371/journal.pone.0213856

**Published:** 2019-03-18

**Authors:** Ayako Sumi, Nobumichi Kobayashi

**Affiliations:** Department of Hygiene, Sapporo Medical University School of Medicine, Sapporo, Hokkaido, Japan; Fundació Institut d’Investigació en Ciències de la Salut Germans Trias i Pujol, Universitat Autònoma de Barcelona, SPAIN

## Abstract

**Background:**

Understanding seasonality of tuberculosis (TB) epidemics may lead to identify potentially modifiable risk factors. Studies conducted outside Japan have found seasonal variation among reported TB cases, with peaks in spring and summer and low prevalence in fall and winter. One hypothesis regarding spring or summer peaks in TB epidemics is that TB transmission likely increases in winter because of indoor crowding and poor ventilation, with development of primary TB among socially vulnerable people in spring and summer. Another plausible explanation is that vitamin D deficiency in winter depresses immunity, increasing the TB reactivation risk in these seasons. Previous studies suggest latitude-dependent factors, including reduced winter sunlight and its effect on vitamin D levels. Here, we investigated mechanisms of seasonality in TB epidemics in Japan, according to the effects of crowding and latitude.

**Methods:**

We used time-series analysis consisting of spectral analysis and least-squares method, to analyse geographically specific monthly number of newly registered cases of all forms of active TB in all 47 prefectures of Japan during 1998–2015.

**Results:**

In all power spectral densities for the 47 prefectures, spectral lines were observed at frequency positions corresponding to a 1-year cycle. The degree of this seasonality was associated with population density. We did not detect greater amplitude of seasonality at higher latitudes, suggesting that latitude-dependent factors, including reduced winter sunlight and its potential effect on vitamin D levels, do not contribute significantly to seasonality in Japan.

**Discussion and conclusion:**

In districts with high population density, measures are needed to address two specific types of active infection risk in adolescents and middle-aged adults: (i) public transport use, and (ii) irregular employment with no periodic medical examinations. To control active TB epidemics, investigating periodic structures in the temporal patterns of active TB in each district and each age group is important.

## Introduction

Understanding the seasonality of tuberculosis (TB) epidemics may lead to identify potentially modifiable risk factors, which might be useful when devising new strategies to identify and treat TB disease and infection and to enhance adherence to therapy [1]. Many studies have examined the seasonality of TB epidemics [2–7]. In an investigation of the periodic structures in age-specific cases of active TB in Japan using a time-series analysis [8], we revealed that occurrence of a seasonal cycle (a 1-year cycle) of active TB epidemics mainly results from epidemic patterns among individuals aged 10–39 years and ≥ 70 years. However, the peak months of active TB epidemics differ between these two age groups; the epidemic peak among individuals aged ≥ 70 years occurs in August and September during the summer, 1–2 months later than the peaks among those aged 10–39 years (in June and July).

Studies conducted outside Japan have similarly found seasonal variation among reported TB cases, with peaks in spring and summer and low prevalence in fall and winter [2–7]. One hypothesis regarding the spring or summer peak in TB epidemics is that the likelihood of TB transmission increases in winter because of indoor crowding and poor ventilation, leading to the development of primary TB among socially vulnerable people in spring and summer [9]. Another plausible explanation is that vitamin D deficiency in winter depresses the immune system, thereby increasing the risk of TB reactivation in spring or summer [7,9,10]. Previous studies have suggested latitude-dependent factors, including reduced winter sunlight and its potential effect on vitamin D levels [5]. The mechanisms of spring and summer peaks of TB epidemics in Japan, including the effects of crowding and latitude, have not been investigated.

TB surveillance data on geographically specific cases of TB in Japan have been collected since 1998 via a nationwide Internet-based infectious disease reporting system. Investigation of the seasonality of these geographically specific TB data could assist with identification of the mechanism of seasonality in TB epidemics in Japan, including the effects of crowding and latitude on disease epidemics. Furthermore, such investigation could facilitate the prediction of epidemics in each geographical area and could improve the incidence of newly registered cases in Japan, which was 15/100000 inhabitants in 2017. The number of newly registered TB cases exceeded 16000 in that year, defining Japan as having a medium TB burden [11]. To clarify the mechanisms of seasonality in TB epidemics in Japan, we designed the present study to investigate the periodic structures of seasonal variations in geographically specific cases of active TB in Japan during 1998–2015, using time-series analysis consisting of a maximum entropy method (MEM) spectral analysis and the least-squares method (LSM) [12,13].

## Methods

### Data

#### Prefecture-specific active TB case data

The modes of detecting active TB in patients in Japan are listed in Table 1. As described in detail previously [8], most patients with active TB are identified in periodic medical examinations (Table 1, section I) or at clinics or hospitals (Table 1, section II). Periodic medical examinations include a chest X-ray, and consist of individual examinations and mass screenings. Mass screenings are conducted for the following population groups, as specified by the TB prevention law: school children and students; inhabitants aged ≥ 65 years; employees in companies, government and municipal offices, schools, hospitals, clinics, birth centres, and social welfare institutions; and inmates of social welfare institutions. If clinical suspicion of TB (Table 1, section II) persists for 2 weeks in individuals diagnosed at a clinic or hospital, a chest X-ray is performed, together with sputum culture if necessary.

**Table 1:** Modes of detection of active TB cases in the study population, Japan, 1998–2015.

	No.	Population (%)
I. Periodic medical examination	88,850	17.5
1. Individual examination	11,253	2.2
2. Mass screening	77,597	15.3
II. Clinic/Hospital examination	408,371	80.3
III. Others/Unknown	11,264	2.2

**Fig 1:**
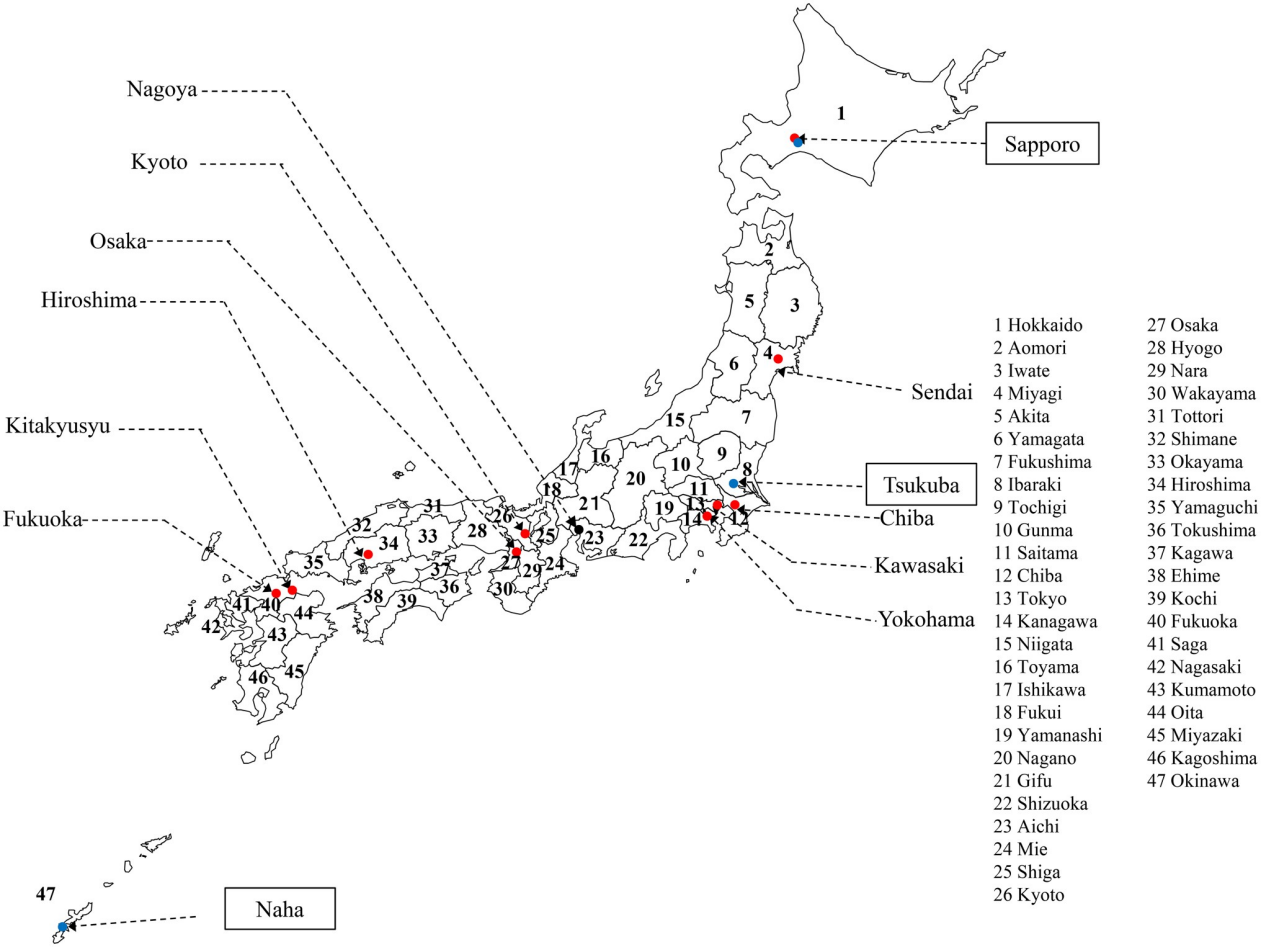
Distribution of the 47 prefectures in Japan. Red circles indicate 12 government ordinance-designated cities with population ≥ 500,000 since 1998: Sapporo, Sendai, Chiba, Yokohama, Kawasaki, Nagoya, Kyoto, Osaka, Kobe, Hiroshima, Kitakyushu, and Fukuoka. Blue circles indicate the three cities where monthly data of the ultraviolet index are collected by the Japan Meteorological Agency: Sapporo, Tsukuba, and Naha in Hokkaido, Ibaraki, and Okinawa prefectures.

The time-series data analysed in this study represent the monthly number of newly registered cases of all forms of active TB for all 47 prefectures in Japan. In Japan’s nationwide surveillance system for infectious diseases, TB surveillance data are collected for all 47 prefectures. The country is divided into prefectures, each of which is further subdivided into cities with respective wards and blocks. The data are available from *Statistics TB* [14] and from the website of the National Institute of Infectious Diseases [15] and are indicated in S1 Dataset. The data for each prefecture were gathered over 216 months (216 data points) from January 1998 to December 2015. The 47 prefectures in Japan are shown in Fig 1.

We also used the monthly number of newly registered cases of all forms of active TB for 12 government ordinance-designated cities in which the population size has been ≥ 500,000 since 1998 [14,15]. These 12 cities are shown in Fig 1. The data are indicated in S1 Dataset.

#### UV index

We used data of the ultraviolet (UV) index, an international standard measurement of the strength of sunburn-producing UV radiation at a particular place and time. In Japan, monthly UV index data are collected in three cities (Fig 1): Sapporo in Hokkaido Prefecture, Tsukuba in Ibaraki Prefecture, and Naha in Okinawa Prefecture. Monthly UV index data were obtained for a total of 132 months from 2005 to 2015 (132 data points) from the website of the Japan Meteorological Agency [16]. The data are indicated in S1 Dataset.

We calculated yearly mean data for both monthly data of active TB in the three prefectures (Hokkaido, Ibaraki, and Okinawa) and the UV index for three cities in these prefectures (Sapporo, Tsukuba, and Naha, respectively) during 2005–2015. We derived the yearly mean number of active TB notifications per 100,000 population using midpoint population denominator estimates for each prefecture taken from the census in each year. The yearly mean data for active TB thus obtained were compared with the yearly UV index data.

#### Time series analysis

The periodic structure of seasonal variations in the monthly time-series data were investigated as previously described [8, 17].

#### Spectral analysis

We assumed that the time-series data *x*(*t*) (where *t* = time) were composed of systematic and fluctuating parts [18]:

x(t)=systematicpart+fluctuatingpart. (1)

To investigate the temporal patterns of *x*(*t*) in the monthly time-series data, we performed an MEM spectral analysis [19]. This method of analysis allows elucidation of periodicities in a time-series of short data lengths with a high degree of frequency resolution compared with other analysis methods of infectious disease surveillance data, such as the fast Fourier transform and autoregressive methods, which require time-series of long data lengths [13]. The MEM spectral analysis produces a power spectral density (PSD). The formulation of MEM-PSD has been described in S1 Appendix.

#### LSM

The validity of the MEM spectral analysis results was confirmed by calculation of the least-squares fitting (LSF) curve to the original time-series data *x*(*t*) with MEM- estimated periods. The formulation of the LSF curve in the *X* (*t*) is described as follows:

$$X(t)=A_{0}+\sum_{n=1}^{N}A_{n}\text{cos}\left\{2\pi f_{n}\left(t+\theta_{n}\right)\right\},$$ (2)

which is calculated using the LSM for *x*(*t*) with unknown parameters *f*_*n*_, *A*_0_, and *A*_n_ (*n* = 1, 2, 3, …, *N*), where *f*_*n*_ (= 1/*T*_*n*_; *T*_*n*_ is the period) is the frequency of the *n*-th component; *A*_0_ is a constant that indicates the average value of the time-series data; *A*_*n*_ and *θ*_*n*_ are the amplitude and phase of the *n*-th component, respectively; and *N* is the total number of components. The reproducibility level of *x*(*t*) by the optimum LSF curve was evaluated by Pearson correlation (*ρ*) with SPSS (Statistical Package for the Social Sciences) version 17.0J software (SPSS, Japan). A *P*-value of ≤ 0.05 was considered the criterion for statistical significance.

#### Contribution ratio

For the assignment of periodic modes constructing the seasonality of the original time-series data *x*(*t*), a ‘contribution ratio’ was defined [8,20]. The contribution ratio *Q*_*n*_ is described as follows:

Qn=An2Q, (3)

where *A*_*n*_ indicates the amplitude of the *n*-th periodic mode constituting the LSF curve *X*(*t*) to the original data *x*(*t*) [Eq (2)], and *Q* is the total power of *x*(*t*). An outline of the contribution ratio is described in S2 Appendix.

#### Outline of the analysis procedure

First, MEM spectral analysis was carried out, and the long-term period was determined from the PSD for the monthly time-series data. Next, the long-term trend in the data were calculated using the LSF method [Eq (2)] with the MEM-estimated period. This LSF curve, corresponding to the long-term trend, was removed by subtracting the LSF curve from the data, and the residual time-series data are thus obtained. Third, the MEM-PSDs of the residual time were calculated. Forth, the seasonality of active TB epidemics was investigated with contribution ratios [Eq (3)] for periodic modes of the residual data.

## Results

### Demographic characteristics of active TB cases

There were 508,485 newly registered cases of active TB in Japan between January 1998 and December 2015, involving more men (64%) than women (36%). The age distribution of the reported cases was as follows: infants and pre-teens aged 0–9 years (0.3%), teens and adults aged 10–24 years (4.5%), adults aged 25–69 years (49.6%), and adults aged ≥ 70 years (45.6%). The proportions of total patients with active TB who were identified during a periodic health examination for TB or at clinics and hospitals were 17.5% or 80.3%, respectively (Table 1).

### Setting up time-series data for prefecture-specific cases of active TB

**Fig 2:**
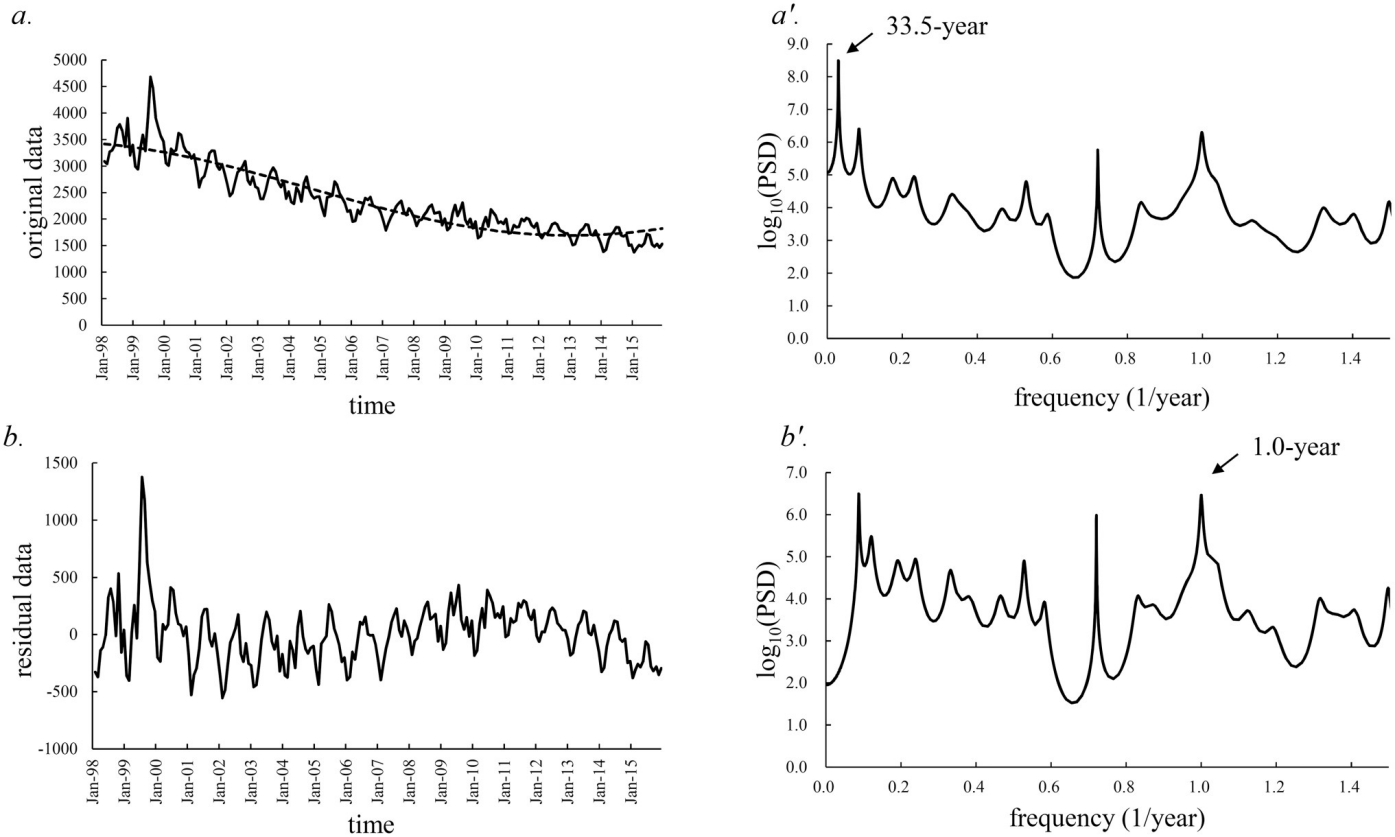
Procedure of the present method of analysis; data for all of Japan are used to briefly explain the method. (a) Monthly number of newly registered cases of all forms of active TB in all of Japan between January 1998 and December 2015 (solid line), and long-term trend calculated with a 33.5-year cycle (dashed line). (a′) Semi-log scale plots of power spectral density [*f* ≤ 1.5 (1/year)], calculated from active TB data for all of Japan. (b) Residual time-series data obtained by subtracting the long-term trend in the active TB data from the active TB data. (b′) Semi-log scale plots of power spectral density [*f* ≤ 1.5 (1/year)] calculated from the residual time-series data.

Fig 2a indicates the monthly number of newly registered cases of active TB in all of Japan during 1998–2015. These data were obtained by summation of the data of all 47 prefectures, shown in S1 Fig.

The PSD, *P* (*f*) (*f* [1/year]: frequency), was calculated for the time-series data (Fig 2a), and the results are shown in Fig 2a′ (*f* ≤ 1.5). In the figure, the longest period appears as a prominent peak at a frequency position longer than the length of the original data (18 years, from January 1998 to December 2015), i.e., a 33.5-year period (Fig 2a). Using this 33.5-year period, the long-term trend in the data was estimated with the LSF using Eq (2); the results are shown in Fig 2a. As shown in the figure, the LSF curve reproduced the long-term trends in the original data well. The residual data are shown in Fig 2b.

### Contribution ratio of the 1-year periodic mode

The PSD was calculated for the residual data (Fig 2b), and the semi-log scale plot (*f* ≤ 1.5) is shown in Fig 2b′. In the PSD, a prominent spectral peak occurs at *f* = 1.0 (= *f*_1_), corresponding to a 1-year period, i.e., the seasonal cycle of disease epidemics. The PSDs of the residual data for all 47 prefectures were calculated, and dominant spectral lines were observed at *f*_1_. Based on this result of the PSDs, we calculated the *Q*_1_ values for all 47 prefectures.

#### Q_1_ value versus population density

**Fig 3:**
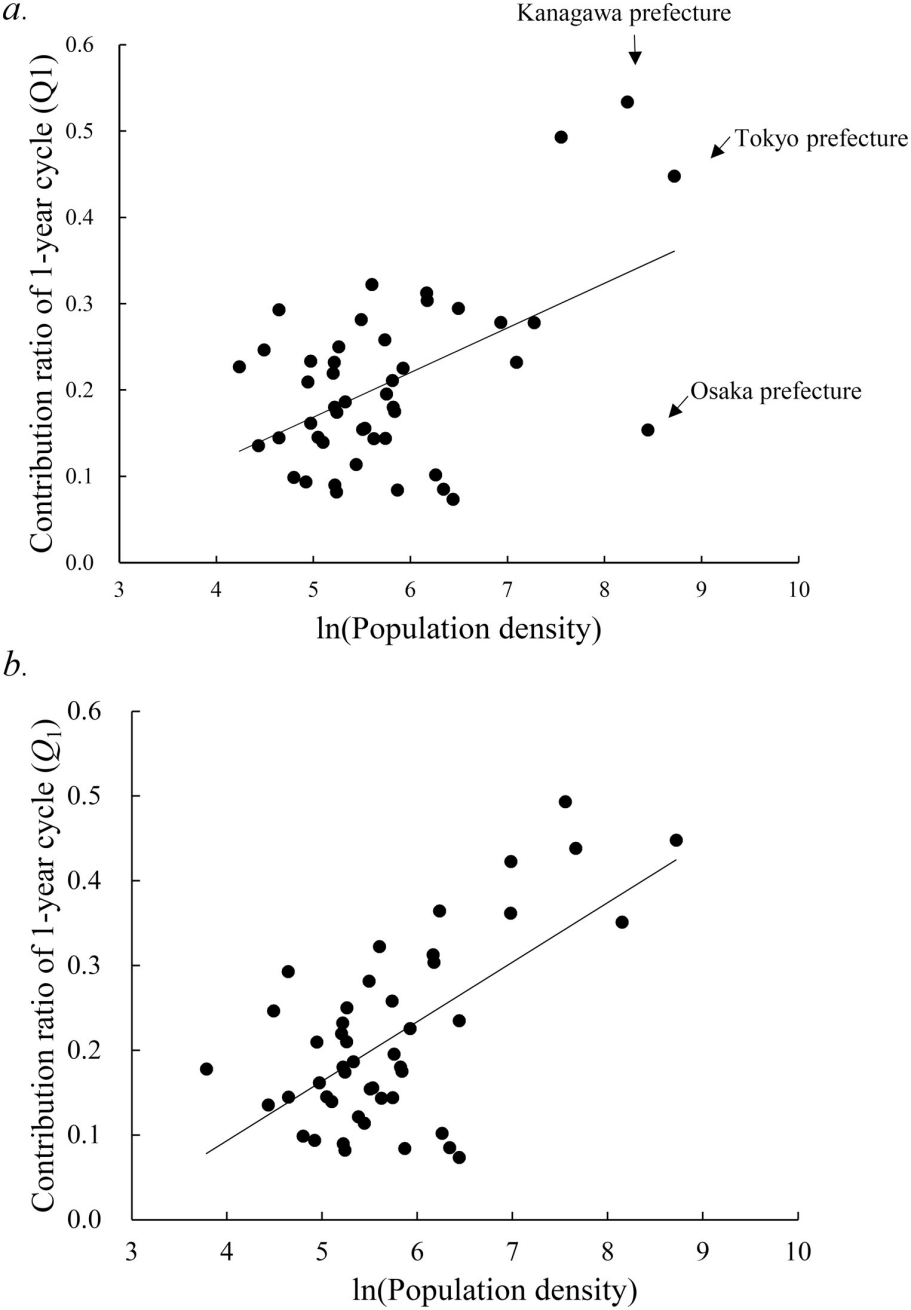
Gradient of *Q*_1_ against the log-transformed population density in the 47 prefectures of Japan from 1998 to 2015. (a) *Q*_1_ obtained from residual time-series data of the 47 prefectures. (b) *Q*_1_ obtained from 35 prefectures and 12 prefectures, which subtracted the share of the government ordinance-designated cities.

We plotted the values of *Q*_1_ against the population density of the 47 prefectures (Fig 3a). The value of *Q*_1_ appeared to increase as the population density increased, although some scattering of points was observed, for example, in Tokyo and Osaka prefectures. The values of *Q*_1_ showed significant correlations with population density (*ρ* = 0.52, *P* < 0.001). This result indicates that occurrence of the unimodal cycle of reported active TB cases in Japan is related to the population density.

We recalculated the value of *Q*_1_ for 12 prefectures, which subtracted the share of the government ordinance-designated cities, and we replotted the obtained *Q*_1_ value against the population density (Fig 3b). The value of *Q*_1_ appeared to increase as the population density increased, and the number of points scattered decreased (Fig 3b). The value of *Q*_1_ showed significant correlation with population density (*ρ* = 0.63, *P* < 0.001). We confirmed that the correlation of *Q*_1_ with population density for the monthly number of TB cases (Fig 3) was also observed for monthly incidence rate of TB cases.

#### Q_1_ value versus latitude

**Fig 4:**
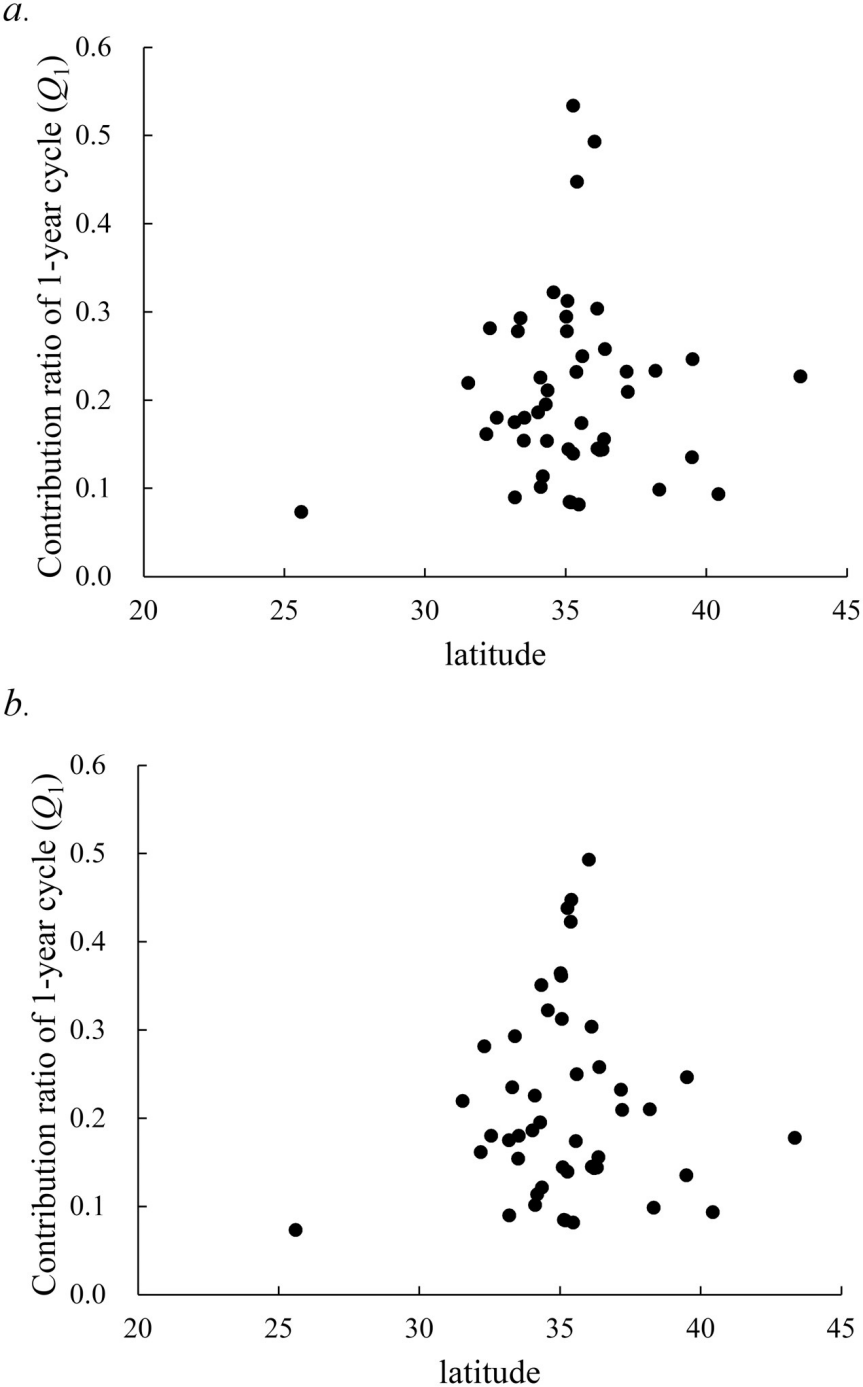
Gradient of *Q*_1_ against the latitude in the 47 prefectures of Japan from 1998 to 2015. (a) *Q*_1_ obtained from residual time-series data of the 47 prefectures. (b) *Q*_1_ obtained from 35 prefectures and 12 prefectures, which subtracted the share of the government ordinance-designated cities.

Fig 4a shows plots of the value of *Q*_1_ against the latitude of the 47 prefectures. The values of *Q*_1_ showed no significant correlation with latitude (*ρ* = 0.076, *P* = 0.613). This result indicates that occurrence of the unimodal cycle of reported active TB cases in Japan has no relationship with the latitude.

We recalculated the value of *Q*_1_ for the residual data of 12 prefectures, which subtracted the share of the government ordinance-designated cities, and we replotted the values of *Q*_1_ against the latitude in Fig 4b. The values of *Q*_1_ showed no significant correlation with latitude (*ρ* = 0.036, *P* = 0.786). We confirmed that the correlation of *Q*_1_ with latitude for the monthly number of TB cases (Fig 4) was also observed for the monthly incidence rate of TB cases.

### TB incidence data and UV index data

**Fig 5:**
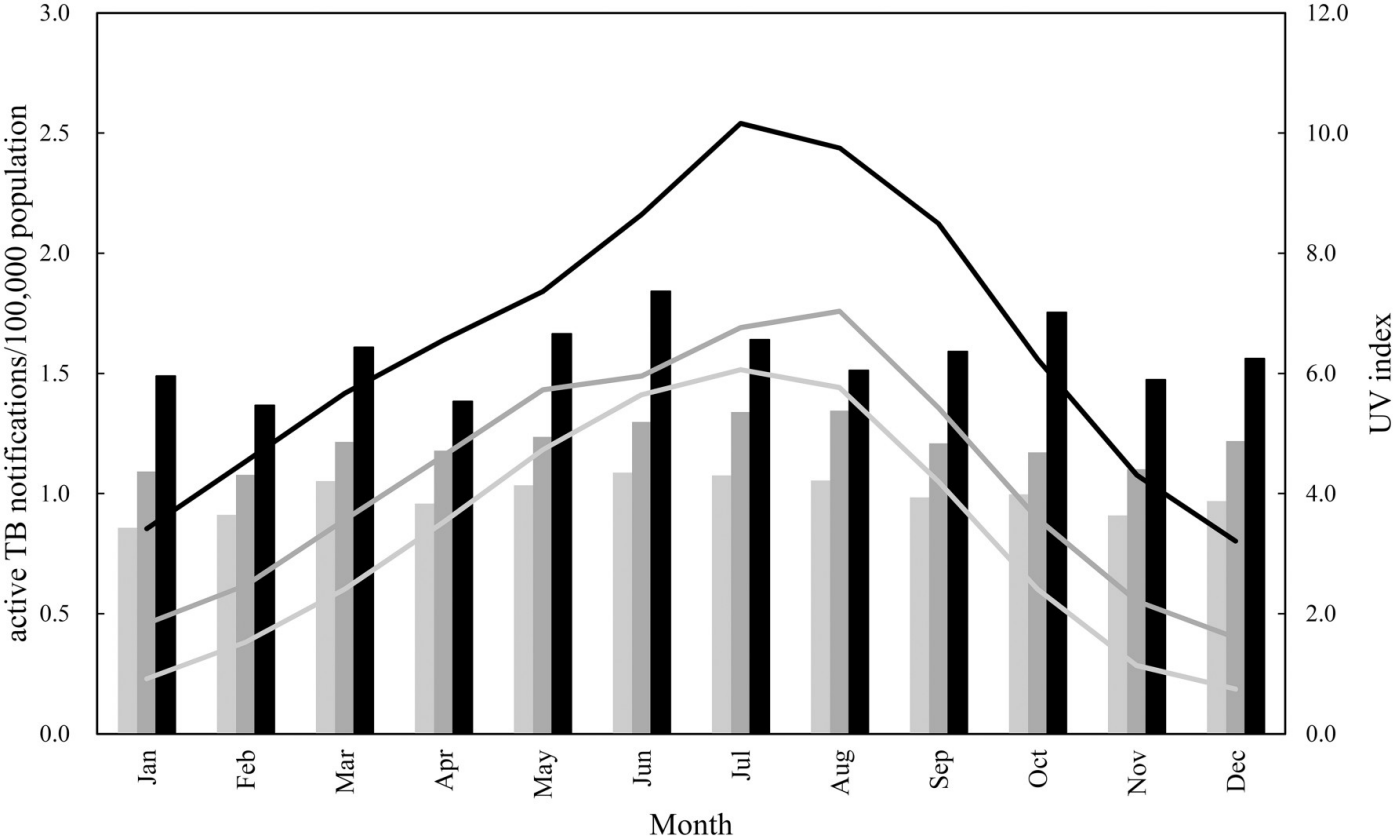
Tuberculosis notifications per 100000 population (bars) and ultraviolet (UV) index (lines) in Japan, 1998–2015. Black bar and line: south region (Okinawa Prefecture and Naha City, respectively); dark grey bar and line: central region (Ibaraki Prefecture and Tsukuba City, respectively); light grey bar and line: north region (Hokkaido Prefecture and Sapporo City, respectively).

In Fig 5, the yearly number of active TB notifications per 100,000 population for Hokkaido, Ibaraki, and Okinawa prefectures were compared with the yearly UV index data for cities in those prefectures, namely, Sapporo, Tsukuba, and Naha, respectively. As can be seen, the value of the UV index for Naha, which has the lowest latitude among the three cities (26°N), was the largest among the three cities over a 1-year period, followed by Tsukuba and Sapporo in ascending order of latitude (36°N and 43°N, respectively). Regarding the yearly mean data for active TB in Fig 5, the value of the data for Okinawa Prefecture (26°N) was the largest among the three prefectures over a 1-year period, followed by Ibaraki and Hokkaido, in ascending order of latitude (36°N and 43°N, respectively); this was also the case for the UV index value.

## Discussion

In this study, we found that the degree of seasonality of active TB epidemics was significantly associated with population density (Fig 3a and 3b). We did not detect greater amplitude of seasonality at higher latitudes, suggesting that latitude-dependent factors, including reduced winter sunlight and its potential effect on vitamin D levels, do not appear to contribute significantly to seasonality in Japan (Fig 4a and 4b). This indicates that vitamin D deficiency, resulting from limited sunlight exposure, as the cause of the spring peak of active TB epidemics is not applicable to the three prefectures of Hokkaido, Ibaraki, and Okinawa (Fig 5). Authors of other studies on the seasonality of TB in India and South Africa have instead suggested that increased active TB disease transmission in winter may occur, owing to greater indoor crowding in colder weather [7,21]. However, seasonal patterns of indoor crowding are unlikely to be uniform throughout Japan, as considered for the United States [2]. Thus, if seasonality of active TB in Japan can be explained by increased transmission in winter, the mechanism may not be as simple as increased indoor crowding, as well as the case of the United States [2].

According to Japanese population censuses, the value of the ratio of active TB cases for individuals aged 10–39 years to all active TB cases increased as the population density (2006 census) increased in Japan (S2 Fig). On the other hand, in the age group ≥ 70 years, the value of this ratio decreased as the population density increased (S3 Fig). Thus, the present result that *Q*_1_ values of active TB cases varied according to population density (Fig 3a and 3b) might be related to environmental and/or biological conditions of individuals aged 10–39 years. As described in detail previously [8], in this age group, there are two specific types of active TB infection risk: (i) the use of public transport [22], and (ii) the situation of young people with irregular employment who do not undergo periodic medical examinations [15]. As future measures, to address the first type of risk, (i), improving early detection of TB in the workplace is concretely recommended; timely examinations at medical institutions with the occurrence of symptoms, thorough countermeasures after periodic medical examinations, thorough examinations for high-risk individuals, and thorough investigation of contacts in the workplace should be considered [8]. With respect to the second type of risk, (ii), conducting thorough periodic medical examinations for people with irregular as well as regular employment should be considered.

A limitation of this study was that we did not have access to data of the UV index for 44 of the 47 prefectures in Japan, only three prefectures (Hokkaido, Ibaraki, Okinawa), as in Fig 5. Increased awareness of the correlation of sunlight with vitamin D levels would result in efficient estimation of the effect of sunlight exposure on TB infections in the remaining 44 prefectures, so as to prevent TB in Japan.

## Conclusion

We identified significant correlation between the degree of seasonality in the geographically specific monthly number of newly registered cases of active TB and population density in Japan (Fig 3a and 3b). To control active TB epidemics, it is necessary to investigate periodic structures in the temporal patterns of active TB in each district as well as each age group, as in our previous study [8]. We anticipate that the present method of time-series analysis, including MEM spectral analysis and LSM, will be useful in further studies of the seasonality of geographically specific TB.

## Supporting information

S1 Dataset Time-series data of the monthly number of newly registered cases of all forms of active tuberculosis in all 47 prefectures and 12 government ordinance-designated cities of Japan and the monthly ultraviolet index data in the three cities of Japan (Sapporo, Tsukuba and Naha). (ZIP)

S1 Appendix Maximum entropy method spectral analysis. (DOCX)

S2 Appendix Determination of the ‘contribution ratio’. (DOCX)

S1 Fig Time-series data of the monthly number of newly registered cases of all forms of active tuberculosis in all 47 prefectures of Japan. (DOCX)

S2 Fig Ratio of active tuberculosis cases in the age group 10–39 years to all active tuberculosis cases. (DOCX)

S3 Fig Ratio of active tuberculosis cases in the age group ≥70 years to all active tuberculosis cases. (DOCX)
