# Supplementary material for: Time-series analysis of geographically specific monthly number of newly registered cases of active tuberculosis in Japan
Source: PLoS One. 2019 Mar 18;14(3):e0213856. doi: 10.1371/journal.pone.0213856 (PMC6422277; doi:10.1371/journal.pone.0213856)
Supplement: S1 Appendix — (DOCX) [file pone.0213856.s002.docx]

# **S1 Appendix. MEM spectral analysis**

Power spectral density (PSD) based on maximum entropy method (MEM), *P*( *f* ) (where *f* represents frequency), for the time series with equal sampling interval ∆*t*, can be expressed by

$P\left(f\right)=\frac{P_{m}\Delta t}{\left|1+\sum_{k=-m}^{m} \gamma_{m.k}\exp\left[-i2\pi fk\Delta t\right]\right|^{2}},$ (A1)

where the value of *P_m_* is the output power of a prediction-error filter of order *m* and *𝛾_m, k_* is the corresponding filter order. The value of the MEM-estimated period of the *n*th peak component *T_n_* ($=\frac{1}{f_{n}}$; where *f_n_* is the frequency of the *n*th peak component) can be determined by the positions of the peaks in the MEM-PSD.
