# Supplementary material for: Time-series analysis of geographically specific monthly number of newly registered cases of active tuberculosis in Japan
Source: PLoS One. 2019 Mar 18;14(3):e0213856. doi: 10.1371/journal.pone.0213856 (PMC6422277; doi:10.1371/journal.pone.0213856)
Supplement: S2 Appendix — (DOCX) [file pone.0213856.s003.docx]

# **S2 Appendix. Determination of the ‘contribution ratio’.**

Based on the result of MEM spectral analysis, we assign periodic modes *f_n_* in Eq. (2) that construct seasonal variations of the original data *x*(*t*) [Eq. (1)]. First, the power of each periodic mode is evaluated by the square of amplitude, *A_n_*^2^, of the *n*-th mode constituting the LSF curve. Second, we estimate *R* corresponding to the power of residual time series, which is obtained by subtracting the LSF curve from the original time series. As a result, the total power of the original time series *Q* is obtained by

$Q=A_{n}^{2}+R.$ (A2)

When both sides of Eq. (A2) are divided by *Q*, we obtain the following normalized relationship:

$\frac{A_{n}^{2}}{Q}+\frac{R}{Q}\begin{matrix} =1 & \end{matrix}$ (A3)

where $\frac{A_{n}^{2}}{Q}$and $\frac{R}{Q}$ correspond to the contribution of $A_{n}^{2}$and *R* to *Q*, respectively. We define the first term of the left-hand side of Eq. (A3) the ‘contribution ratio’, which means the contribution $A_{n}^{2}$normalized by *Q*. If $\frac{A_{n}^{2}}{Q}$in the first term becomes large, then the second term, $\frac{R}{Q}$, becomes small.
