# Supplementary material for: Time-series analysis of geographically specific monthly number of newly registered cases of active tuberculosis in Japan
Source: PLoS One. 2019 Mar 18;14(3):e0213856. doi: 10.1371/journal.pone.0213856 (PMC6422277; doi:10.1371/journal.pone.0213856)
Supplement: S1 Fig — (DOCX) [file pone.0213856.s004.docx]

# **S1 Figure. Time-series data of the monthly number of newly registered cases of all forms of active tuberculosis for all 47 prefectures of Japan.**

**
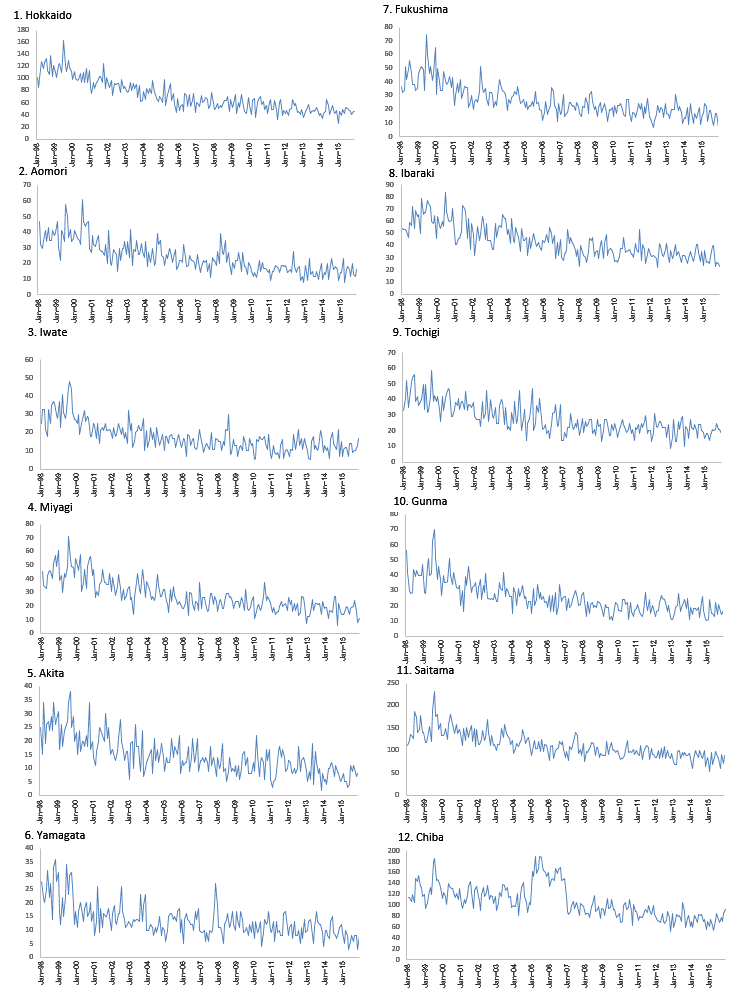
**

# **S1 Figure (continued)**


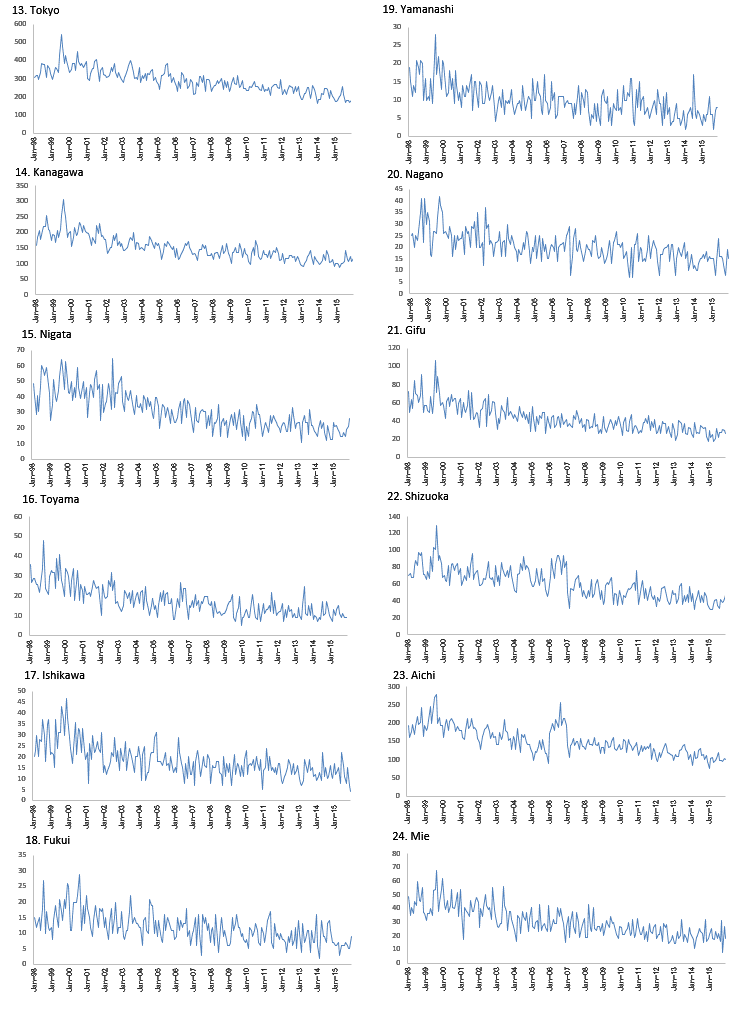


# **S1 Figure (continued)**

**
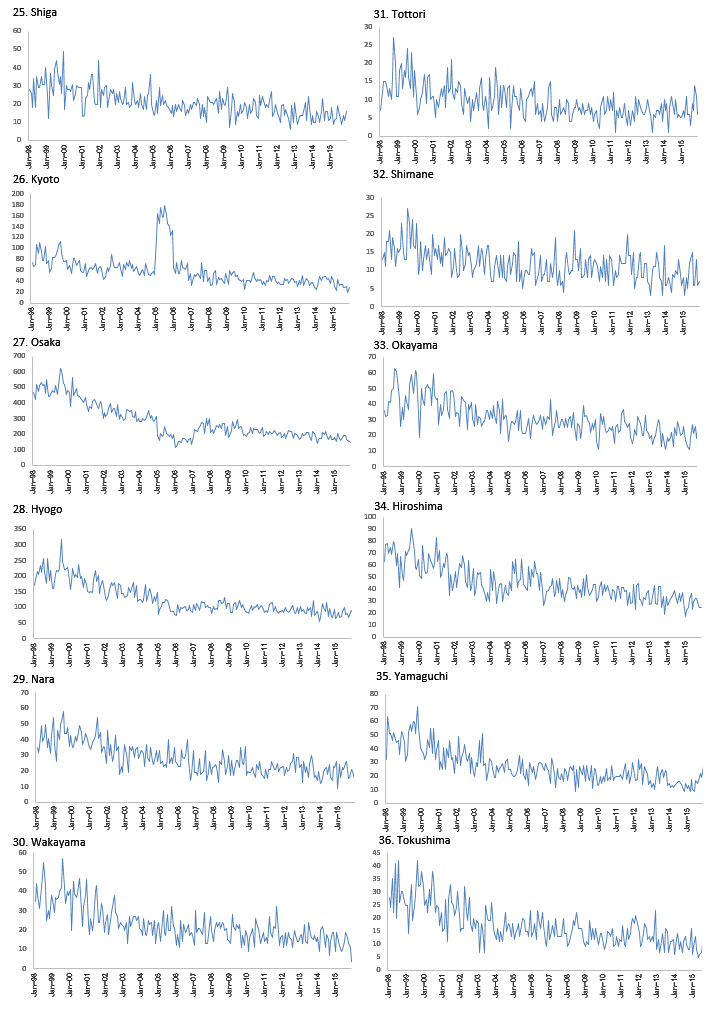
**

# **S1 Figure (continued)**

**
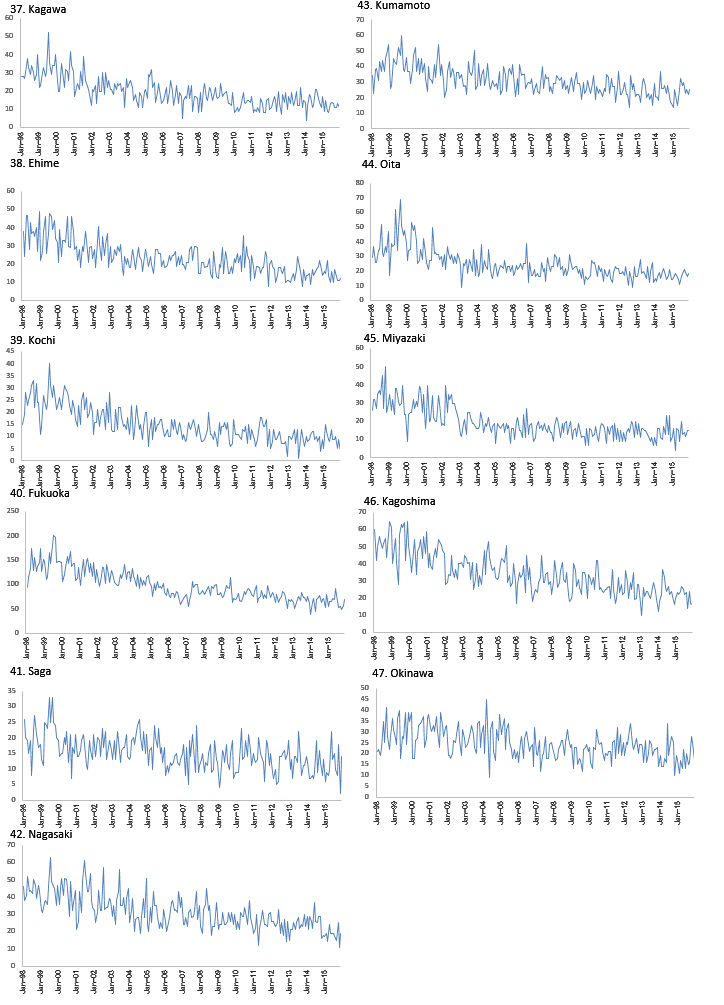
**
