# Supplementary figures and images for: Time-series analysis of geographically specific monthly number of newly registered cases of active tuberculosis in Japan
Source: PLoS One. 2019 Mar 18;14(3):e0213856. doi: 10.1371/journal.pone.0213856 (PMC6422277; doi:10.1371/journal.pone.0213856)

# **S2 Figure: Ratio of active TB cases in the age group 10–39 years to all active TB cases.**

**
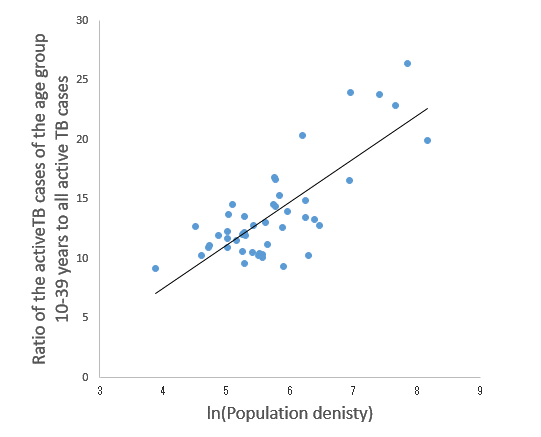
**

Supplement: S2 Fig — (DOCX) [file pone.0213856.s005.docx]

# **S3 Figure. Ratio of active TB cases in the age group ≥70 years to all active TB cases.**

**
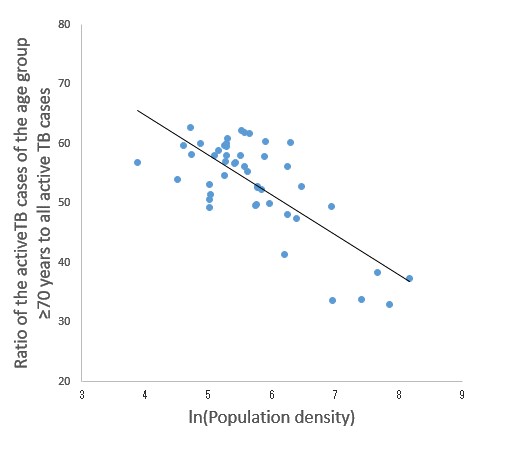
**

Supplement: S3 Fig — (DOCX) [file pone.0213856.s006.docx]
